# Is Ejaculation Frequency in Men Related to General and Mental Health? Looking Back and Looking Forward

**DOI:** 10.3389/fpsyg.2021.693121

**Published:** 2021-08-09

**Authors:** Anna Mascherek, Mirjam Christina Reidick, Jürgen Gallinat, Simone Kühn

**Affiliations:** Department of Psychiatry and Psychotherapy, University Medical Center Hamburg-Eppendorf, Hamburg, Germany

**Keywords:** ejaculation frequency, general and mental health, moral incongruence, quality of sperm, brain structure and function, modifiable lifestyle behavior, abstinence

## Abstract

Despite its relevance for human sexuality, literature on potential effects of ejaculation frequency and masturbation on general and mental health outcomes is sparse. Reasons for this knowledge gap include a general lack of interest, but also methodological challenges and still existing superstition. This paper reconciles literature from various fields to extract relevant information on how ejaculation frequency effects general and mental health outcomes. Culture-bound syndromes have been reported in countries still strictly tabooing or condemning masturbation. Masturbatory guilt describes a phenomenon in individuals experiencing a discrepancy between moral standards and own behavior with respect to masturbation. Abstinence is one aspect under study in the area of fertility treatment. Specific time frames and their respective implications on quality of sperm remain inconclusive. Limited temporal resolution capacities hamper the precise study of brain structures directly activated during ejaculation. The relation between ejaculation frequency and hormonal influences remains poorly understood. Future research that specifically addresses ejaculation frequency and potential mental and general health outcomes is needed. In contrast to extracting knowledge as a byproduct from other studies with a different focus, this enables sound study designs and could provide evidence-based results which could then be further discussed and interpreted.

## Introduction

A growing body of research and campaigns promote masturbation as safe self-sex behavior and no ill effects of masturbation have been reported up until today. Still, online communities promoting abstinence from masturbation are on the rise with a steadily growing number of followers. The subreddit “NoFap” and its accompanying website (NoFapp LLC, 2020) has currently more than 738,000 followers. It represents an online community where members challenge themselves to abstain from pornography and masturbation to strengthen mental and physical health, (re)gain self-confidence, increase productivity by raising energy levels, and improve social (and romantic) interactions (Fernandez et al., 2021). Some of the more radical points of view within the community even promote abstinence from orgasm in general, not only from masturbation, as a healing experience. The most interesting aspect of such internet-based, social-media movements is the widespread notion that masturbation as such is subject to negative appraisal (Hartmann, 2020). The motivation to abstain is rooted in the belief that masturbation is unhealthy (Zimmer and Imhoff, 2020). And while hypersexuality and excessive pornography consumption have been acknowledged as problematic behavior (Kafka and Hennen, 2003; Kafka, 2010; Grubbs et al., 2019a, b), this is not scientifically established for the effect of masturbation and ejaculation on general or mental health at all.

In the present paper, we discuss literature from different fields that have addressed the potential relation between ejaculation frequency^1^ and general and mental health outcomes. We will touch on different domains such as abstinence, quality of sperm, and lifestyle variables. The overarching aim of the present study is to elucidate potential knowledge gaps. We discuss reasons to conduct research to fill those gaps rather than presenting an in-depth description of the detailed study situation in every field. Many studies cited in the present paper do not primarily focus on the potential relation between ejaculation frequency and general and mental health. Our topic of interest is often marginally referenced when addressing the actual research question of the respective study. However, in reconciling those side-aspects from different studies and even from different disciplines, a picture emerges revealing open questions for future studies. To get a solid overview of the literature, two of the authors independently conducted a comprehensive literature search. The search ended when no new records could be found and also no additional information could be extracted from the references, leaving the study team with a sound overview over the relevant literature. We believe that our endeavor is a valid concern as ejaculation frequency represents a central aspect of the sexual response cycle. It is, however, not strictly limited to solely reproductive purposes, e.g., when thinking of masturbation, but can be considered a modifiable lifestyle variable. Taking this perspective, surprisingly little is known about ejaculation frequency and potential implications for general and mental health. For a comprehensive summary of the points discussed below, see Table 1.

**TABLE 1:** Summary of positions presented and empirical questions to be addressed.

**Position**	**General understanding from the literature**	**Empirical research questions**
**Masturbation and ejaculation**		

…and the historical perspective	Ejaculation and masturbation have a long history of superstition. Perspectives on masturbation have changed from representing a harmful and deviant behavior to being perceived as normal and even healthy. However, this general notion varies depending on group membership. Large differences exist between countries, becoming evident in so-called “culture-bound syndromes.” Differences in attitude also run along group membership holding different value systems, educational levels, or professions.	Which factors exert systematic and significant impact on societal attitudes toward masturbation and ejaculation frequency. Is there an effect of societal attitudes on attitudes toward masturbation and ejaculation on individual level? Can those effects be categorized or explained?
…and quality of sperm	Sperm quality is described along six parameters (WHO spermiogram). Abstinence as influential factor has repeatedly been described, however, without clear time specifications.	Does ejaculation frequency significantly impact quality of sperm? If ejaculation frequency impacts quality of sperm, which are the parameters mostly affected? Is the effect of ejaculation frequency different for ejaculation due to masturbation or sexual intercourse?
…and hormonal influences	Human male sexuality is influenced by a complex interplay of biomarkers, predominantly by testosterone. The focus of literature lies on the causal direction. Little is known on the effects of ejaculation and masturbation frequency on testosterone levels.	Are there potential associations between different biomarkers and frequency? How do ejaculation/masturbation frequency and testosterone levels affect each other in the long run? Does ejaculation/masturbation abstinence significantly affect testosterone levels?
… and brain function and structure	Limited temporal resolution of imaging techniques and other context factors hamper the precise study of processes directly involved in ejaculation and orgasm.	Which brain structures are activated during ejaculation? What effects does frequent ejaculation have on typically involved structures and their function? Does a thorough understanding of the ejaculation process aid in the understanding and treatment of sexual dysfunctions?
…and modifiable lifestyle variables	Mental and general health and well-being are subject to different behaviors subsumed under the term “modifiable lifestyle factors.” Masturbation and ejaculation frequency have not been listed, however, represent modifiable, individual, and presumably influential factors regarding well-being. Some studies argue that not the behavior itself but rather accompanying attitudes and potential discrepancies between attitudes and behavior might exert the influence on well-being.	Is there a relation between masturbation and ejaculation frequency and mental and general health/well-being? Is the potential relation between masturbation and ejaculation frequency and mental and general health/well-being mediated through personal beliefs? Is a potential discrepancy between personal beliefs and individual behavior more important than the actual belief itself?

## Prevalence of Masturbation

Masturbation to orgasm has been scientifically and medically recognized as a common practice among humans across the lifespan (Smith et al., 1996; Meston, 1997; Waite et al., 2009; Robbins et al., 2011). Research on the frequency of masturbation shows that men and women report masturbating regularly, however, with differences in frequency. In an American sample, 38% of women and 61% of men reported masturbatory behavior over the past year (Das, 2007). These numbers are confirmed in a British sample with 33% of women and 66% of men reporting masturbation within the preceding 4 weeks (Mercer et al., 2013). Similar numbers are reported for Australia (Richters et al., 2014), implying that the prevalence is high and rather universal. Estimates are also probably conservative due to a potential lack of disclosure. Interestingly, epidemiological studies do not only report differences in frequency as a function of gender and age (Mercer et al., 2013), but also of educational-level, frequency of sexual intercourse, or religious affiliation (Gerressu et al., 2008). It is established that masturbation is a common behavior in both, men and women (Carvalheira and Leal, 2013), with women being even more sparsely covered in the literature. We decided to focus on behavior in men only, as it was beyond the scope of the paper to cover both. However, masturbatory behavior in women is an interesting and widely understudied topic, in particular since it seems to be even more tabooed than masturbatory behavior in men.

Before turning to existing literature, we provide a brief historical summary on social attitudes toward masturbatory practices and historic changes. We do so, because on the one hand, social perception of masturbation faces a centuries-long history of ostracism, and on the other hand, beliefs and attitudes potentially mediate the effects of masturbation on health.

## Historical Perspective on Masturbation

The modern perspective of health professionals implies that masturbation is a positive aspect of healthy sexual development within the range of normal human behavior (Driemeyer, 2013). Masturbation is also an important part of sex therapy (LoPiccolo and Lobitz, 1972; Zamboni and Crawford, 2003), however, not without controversy (Christensen, 1995). Yet, while the scientific community recognizes masturbation as normal and beneficial behavior, the social discourse is still characterized by taboo (Das, 2007; Das et al., 2009; Carvalheira and Leal, 2013). This illustrates a discrepancy between social reality and social appraisal. The glorification of abstinence that is pursued by some social media movements, can be interpreted as a modern version of the historic perspective taken on masturbation.

As early as from the time of Hippocrates, but most prominently from the beginning of the 18th century, masturbation was regarded as deviant and harmful behavior that eventually led to insanity as well as bodily decay (Whorton, 2001). Even nocturnal emission as the involuntary physical reaction was deemed sinful. Loss of semen was generally believed to weaken the male’s body and constitution. While this view has a long-standing history in religious writings, it also came to the notion in medical writings in the late 1700s. It was strongly promoted in the 19th and early 20th century (Bullough, 2003) from both, medical as well as religious and pedagogical perspectives (Stolberg, 2000). The notion that masturbation was sinful and dangerous to body and mind prevailed throughout the 19th century. Historical change started around the turn of the century. The predominant view was more and more labeled as superstitious. It was promoted as unlikely that masturbation caused mental illnesses (Patton, 1986; Whorton, 2001). Alfred Kinsey published his seminal work on sexual practices in America in the mid-1940s (Kinsey et al., 1948). From thereon, at least the scientific perspective changed rapidly. Masturbation today is acknowledged as natural, normal, and even beneficial sexual behavior (Levin, 2007). It is promoted as one safe-sex behavior, preventing sexually transmitted diseases and unwanted pregnancies especially in adolescence (e.g., Robinson et al., 2002). Societal change and official positions are mirrored in documents provided by the World Health Organization (WHO) on sexual education in Europe (WHO Regional Office for Europe and BZgA, 2010). Masturbation practices are described as normal developmental aspects of healthy sexuality and fundamental aspect of sexual education.

An indirect effect of masturbation on mental health has been implied by literature on so-called culture-bound syndromes. In this context, it can be generally subsumed under the heading of semen-loss anxiety. The term culture-bound syndrome is not without controversy itself, as it is suspected of reproducing imperialistic or eurocentristic worldviews. However, it should be understood as a description of a bundle of symptoms that can only be found in specific culturally or socially defined groups. Culture-bound syndromes are not unique to sexual behavior, bulimia nervosa, for example, has been described as a culture-bound phenomenon mainly present in the western hemisphere (Sumathipala et al., 2004). The *dhat-syndrome* is mainly present on the Indian subcontinent and describes a general semen-loss anxiety. The symptoms are predominantly found in young, unmarried Indian men. Symptoms are fatigue, weakness, anxiety, and feelings of guilt (Udina et al., 2013). The symptoms resemble those of affective or anxiety disorders, however, the causal attribution of the symptoms by dhat-patients is semen loss due to nocturnal emissions or masturbation.

A related, yet geographically distinct, syndrome is described by *shen-k’uei* (Sumathipala et al., 2004) in China. Again, loss of semen due to nocturnal emission, frequent intercourse, or masturbation is causally linked to anxiety, weakness, and insomnia by patients. Etiological explanations for the syndrome are rooted in classical Chinese medicine. Similar syndromes were described in western cultures in the 19th century. Mainly based on religious grounds, masturbation was prohibited and ostracized. It was believed that masturbation and also nocturnal emission causes disorders such as weakness, headaches, anxiety, and general physical weakness (Stolberg, 2000). Those beliefs clearly resemble those of *dhat*- and *shen-k’uei-* patients. The strong cultural and societal impact on the development of psychological strain is striking. All syndromes have a profound moral component in common that is paramount for the development of psychological strain and distress. The syndromes can be described as type of anxiety disorder. Up until today, there is no proof of existence of a biological relation between masturbation or nocturnal emissions and any of the described symptoms above (Sumathipala et al., 2004). Nonetheless, awareness of the phenomena is important as they cause real and severe psychological distress in individuals (Ventriglio et al., 2016).

Scientific literature today is not always completely free from reservations toward masturbation (Brody and Costa, 2009; Jiao et al., 2019). However, addressing ideology is difficult. It sometimes lies under scientific veneer, referencing scientific studies (Speed and Cragun, 2018). In other work, ideology is more striking and easier detected (Brody et al., 2012; Hoseini, 2017). There is a corrective lobby for ideological articles (Speed and Cragun, 2018). Especially with a topic inherently prone to bias, it is essential to raise awareness for both the interpretation of existing literature and the conduction of future studies.

Literature is often biased by a “WEIRD”-perspective. Henrich et al. (2010) established WEIRD as an acronym by describing a tendency in psychological science to base results on samples that mainly represent Western, Educated, Industrialized, Rich, and Democratic societies. In their seminal work, Henrich et al. (2010) pointed to the fact that more than 80% of the studies published are based on samples representing only around 12% of the world’s population. He demanded to at least incorporate a critical reflection on sampling in the limitation section of a paper if relying on WEIRD individuals. While Henrich’s work addressed psychological science as a whole, his point especially applies to the understanding of masturbatory practices and attitudes toward masturbation.

## Masturbation and Quality of Sperm

The relation between ejaculation and quality of sperm is a central question in the discussion of potential effects of frequent ejaculation and health. Ejaculation is an essential part of male reproduction. In the context of reproduction and assisted fertility treatment ejaculation is important as a necessary body function. But also, the quality of the resulting semen plays a vital part in the reproductive success of human individuals. Standard procedures for the preparation of a spermiogram are defined in the WHO’s laboratory manual on the analysis of human sperm (World Health Organization (WHO), 2010). A spermiogram evaluates the quality of sperm along the following dimensions: semen volume, sperm concentration, total sperm count per ejaculate, sperm motility, sperm morphology, and sperm vitality. Sperm concentration, motility, and morphology represent the three classical parameters analyzed in nearly all laboratories (Wang and Swerdloff, 2014). Although those parameters with respective reference limits have been acknowledged as guiding principles, it is also well established that the references are not set in stone. Studies showed that depending on measurement technology, but also depending on geographic location and even racial and ethnic affiliation, the references of the parameters vary (Jørgensen et al., 2001; Swan Shanna et al., 2003; Wang and Swerdloff, 2014).

One question that has received considerable attention is whether or not abstinence affects the quality of sperm. This question has mainly been studied in the field of reproductive medicine and infertility. However, it is interesting in its own right to understand the possible relation between ejaculation frequency and health. A recent review (Hanson et al., 2018) summarized the question of whether or not abstinence has an impact on the quality of sperm. Reviewing 28 publications published since the year 2000, the authors conclude that the impact of abstinence on sperm quality is complex and inconclusive. As of today, it remains unclear which parameter ultimately is most important for successful fertilization. There is some evidence that abstinence of less than 3 days is associated with higher pregnancy rates in artificial insemination (Sánchez-Martín et al., 2013; Ayad et al., 2018). Although sperm count and semen volume seem to improve with longer abstinence, this is not certain for motility, vitality, and morphology. Be this either due to study design and assessment method or real findings remains unclear. Some studies conclude that abstinence could be recommended, however, with a plateau being reached after a few days (De Jonge et al., 2004; Levitas et al., 2005; Hanson et al., 2018). The WHO’s general recommendation of 2–7 abstinence days to improve semen parameters is challenged as evidence is inconclusive.

A worldwide trend in a general reduction in the quality of human male sperm parameters is observed (Virtanen et al., 2017), though, again, not without inconsistencies. Explanatory approaches name the effect of biological as well as environmental or lifestyle variables on sperm quality, such as pollution, age, nutrition, and stress (MacDonald et al., 2009; Li et al., 2011; Virtanen et al., 2017; Arab et al., 2018; Durairajanayagam, 2018; Ilacqua et al., 2018). In those studies, so-called modifiable lifestyle factors are examined. Modifiable lifestyle factors refer to behavior that is ultimately controlled by the individual. Modifiable lifestyle factors may be protective as well as harmful. Results of studies are inconclusive with respect to precisely describing the impact of specific lifestyle-variables. Yet, the overarching tone is that lifestyle variables are influential. This is true for more objectifiable variables such as nutrition habits, smoking, or alcohol intake, but also for less obvious aspects such as stress (Ilacqua et al., 2018), or depression and anxiety (Wdowiak et al., 2017). According to our reading, masturbation and ejaculation have generally been neglected as unique lifestyle factors with a potential impact on both sperm quality and general health and well-being. Especially as masturbation comprises a part of the sexual response itself, it could be linked way closer to sperm quality than other rather distant aspects of general lifestyle (e.g., physical activity).

Some studies on quality of sperm suggest that the assessment method should be included as covariate. Methodological aspects have proven influential over and above differences in the sperm itself (Brackett and Lynne, 2000). One study reported that semen samples collected from masturbation at home or the clinic differed significantly with respect to sperm motility, total count, and concentration (Elzanaty and Malm, 2008). From a randomized controlled trial on whether erotic magazines facilitated semen collection, the authors conclude that the context of collecting semen samples should always be taken into account as potentially influential (Handelsman et al., 2013). Other studies report semen samples collected from penile-vaginal intercourse as being higher in quality than semen from masturbation (Zavos and Goodpasture, 1989; Sofikitis and Miyagawa, 1993). These studies altogether underline the potential impact of variables other than the actual specimen. While this might not be decisive in the context of reproductive medicine and fertilization, it is relevant for basic research to understand underlying mechanisms.

Although research shows inconclusive results concerning the medical benefit of abstinence, social movements pursue this idea. The focus often lies on perceived mental and social benefits including better health, increased masculinity, and mental clarity (NoFap LLC, 2016). Scientific evidence is lacking; however, initiators of the movement repeatedly refer to scientific literature, making it difficult to distinguish empirical evidence from ideology.

## Hormonal Influences and Related Biomarkers in the Context of Ejaculation and Masturbation

The male testis has two central functions: spermatogenesis and synthesis as well as secretion of hormones (Amann, 1989). Sexuality is strongly influenced by hormones (Krüger et al., 2003) in particular, by sex hormones including androgens, estrogens, and progesterone (for a review see Meston and Frohlich, 2000). Human male sexuality is predominantly influenced by the androgen testosterone. The testosterone synthesis process starts with the secretion of gonadotropin-releasing hormone in the hypothalamus, which in turn acts on the secretion of the gonadotropins luteinizing hormone (LH) and follicle-stimulating hormone in the anterior pituitary. LH subsequently acts on Leydig cells in the testicles and this leads to testosterone release (Hock, 2016). Next to being involved in spermatogenesis (Nassar and Leslie, 2021), testosterone is predominantly associated with the initiation of sexual arousal and desire (Vignozzi et al., 2008); with higher levels of testosterone often being linked to increased sexual activity and interest (e.g., Carani et al., 2005). Sexual activity itself appears to have a short-term effect on testosterone as far as testosterone levels appear to increase after watching erotic stimuli or penile-vaginal intercourse, as a review shows (Van Anders and Watson, 2006). Additionally, a naturalistic study in a sex club supported that testosterone levels increase temporarily when observing and especially when engaging in sexual behavior (Escasa et al., 2011). Masturbation in particular seems to have the same temporary trend, yet results are limited (Van Anders and Watson, 2006; Escasa et al., 2011). Studies on patients with erectile dysfunction give further support for an influence of sexual activity on testosterone levels (Jannini et al., 1999, 2009; Carosa et al., 2002, 2004). Low or a loss of sexual activity due to erectile dysfunction is accompanied by low testosterone levels; while a resumption of sexual activity appears to restore testosterone levels, irrespective of the cause or treatment of the erectile dysfunction. To note, sexual activity was ascribed as full sexual intercourse and, therefore, no conclusion specifically concerning masturbation can be drawn yet (Jannini et al., 1999, 2009; Carosa et al., 2002, 2004).

Social media sites that promote masturbatory abstinence regularly claim that there is scientific evidence that abstinence is beneficial for men’s testosterone levels. Those assertions are accompanied by claims that promote stronger mental health (Hartmann, 2020). These sites support their statement by referencing a study (Jiang et al., 2003), in which a peak in serum testosterone levels after 7 days of abstinence was reported in male participants. Notably, the results were reported on the basis of a small sample size. To the best of our knowledge, these results were not replicated so far. In this line of research, another study investigated the effect of 3-weeks abstinence on different endocrine responses including testosterone. Here, the authors reported that abstinence led to an increase in basal testosterone level, yet did not alter the typical cardiovascular and endocrine responses to orgasm. Therefore they concluded that abstinence has an insufficient impact on endocrine responses (Exton et al., 2001). To note, this study also had a small sample size. Far-reaching statements about beneficial effects of abstinence on male’s testosterone levels need to be taken with caution. Indeed, the role of testosterone in sexual activity appears to be more nuanced.

In this line of inquiry, a focus lies on identifying the causal direction, meaning whether testosterone levels cause certain sexual behavior (i.e., hormonal causation pattern; Kraemer et al., 1976; Knussmann et al., 1986; Carani et al., 2005; Archer, 2006; Finkelstein et al., 2013) or whether a certain behavior or environment causes testosterone levels to change [i.e., reverse relationship, “social modulation” model (Van Anders and Watson, 2006; Van Anders et al., 2015; Das and Sawin, 2016)]. The causal pattern of testosterone is often studied in connection with sexual activity, relationship commitment, and parenting effort. Das and Sawin (2016), for example, attempted to unravel the causal pattern of the effects of testosterone on frequency of partnered sex and masturbation as well as on relationship quality. They conducted a longitudinal study with a representative United States sample of older, both male and female, adults (aged 57–85). For the male participants, a higher masturbation frequency predicted higher levels of testosterone which gives support for the social modulation model. On the other hand, higher levels of testosterone negatively affected relationship quality later in life, which points to a hormonal causation (Das and Sawin, 2016). Moreover, the connection between ejaculation frequency and testosterone levels might play a role in understanding and explaining ejaculatory dysfunctions (Rastrelli et al., 2018). A low level of testosterone is associated with reduced volume in ejaculation and delayed ejaculation. High levels of testosterone are associated with premature ejaculation (Corona et al., 2008). Premature ejaculation, in turn, may influence orgasmic pleasure. In specific, lower scores on the “Orgasmometer,” a subjective measure for the intensity of an orgasm, are observed for individuals with premature ejaculation (Limoncin et al., 2016). The orgasmometer as a tool has also been used to assess orgasmic intensity in healthy individuals (Mollaioli et al., 2021).

As mentioned, the male testis has two major functions. Next to the secretion of hormones, particularly testosterone, testicles produce sperm (Amann, 1989). Testosterone is just one factor that contributes to spermatogenesis and testicular development. Neurotrophins, growth factors in the nervous system, came to our attention, as they are possibly also involved in testis development and spermatogenesis (for a review of the role of neurotrophins in male reproduction see Li and Zhou, 2013). It is interesting that neurotrophins, which are typically involved in diverse parts of neuronal growth and functions such as differentiation, survival, synaptic plasticity, or apoptosis (Li and Zhou, 2013; Bathina and Das, 2015), appear to be also involved in non-neuronal tissues (Müller et al., 2006; Li and Zhou, 2013). Both, the nerve growth factor (NGF) and the brain-derived neurotrophic factor (BDNF), belong to the group of neurotrophins and have been found in ejaculated sperm. Especially BDNF was proposed as a marker for semen quality (Zheng et al., 2011). In line with this is the finding that treatment of sperm with BDNF increases the motility of sperm, as BDNF is assumed to have protective effects against oxidative stress. Therefore, BDNF has been suggested to improve sperm in order to ultimately help fertilization (Najafi et al., 2017). To note, this area of research is rather limited and we do not want to suggest any association or draw any conclusion of what this means with regard to ejaculation and masturbation frequency in specific. Yet, we want to acknowledge the complex interplay of biomarkers in male sexuality. Along with this and also the next paragraph we want to mention the recent review (Matos et al., 2021) that pointed to a striking similarity between the brain and testis.

Thus, further research is encouraged to generate profound knowledge and aid the understanding of potential relations, for instance that NGF possibly mediates the effects of testosterone in spermatogenesis (Li and Zhou, 2013).

## Ejaculation, Masturbation, and Brain Function and Structure

Ejaculation and orgasm are very brief, time-limited actions of human male sexual behavior. A large body of research deals with the brain’s functional and structural setup and functional connectivity within the human sexual response (Poeppl et al., 2014; Ruesink and Georgiadis, 2017). Comprehensive reviews on the broader picture of human male sexual response with respect to brain imaging can be found elsewhere. Generally, broader aspects of the sexual cycle (wanting, liking, ad inhibition) are under study [see (Georgiadis et al., 2012) for an extensive review]. Only few studies exist that directly analyze brain activation during orgasm, because imaging techniques are hampered by their temporal resolution capacities.

Two studies from the same group vividly illustrate the methodological difficulties. Georgiadis et al. (2007) analyzed brain activation during ejaculation in a positron emission tomographic study. They report decreased activity in prefrontal cortex areas, supporting the notion that the prefrontal cortex exerts inhibitory control over sexual functions. The authors also report increased activity, most prominent in the left dentate nucleus within the cerebellum and the ventrolateral part of the transition zone of midbrain and thalamus. The results described above came from a re-analysis of data previously published by the same group (Holstege et al., 2003). The re-analysis led to more precise results due to an improved signal-to-noise ratio. The authors concluded that the refinement led to more precise images of human male brain activity during ejaculation. Results from the first analyses were classified as artefactual. The authors critically discuss that, simply due to a refined analysis-technique, activation in the striatum, the midbrain, the thalamus, the cerebellar hemispheres, and parts of the neocortex could no longer be attributed to the human male ejaculatory process. Although those regions are all involved in the sexual response, they were not directly linked to the ejaculatory process.

Sexuality, in general, is a well-established subject in research, but this is less the case for the specific aspect of human male ejaculation. This also applies to research on neural correlates in patients with lifelong premature ejaculation. Some studies reported decreased brain activity in the left inferior frontal gyrus and left insula (Zhang et al., 2017; Yang et al., 2018). But those results refer to general differences in activation between a clinical and a healthy sample. Research on brain activity specifically during ejaculation would enable the understanding of potential impact of frequent ejaculation on brain structure and function. Knowledge here could enable the deeper understanding of mechanisms underlying sexual dysfunctions.

## The Relation Between Masturbation and Ejaculation and Well-Being as Well as Lifestyle

Contrasting its prevalence, surprisingly little is known about the impact of ejaculation and masturbation on mental health and well-being (but see Brody, 2006, 2010; Levin, 2007). Human sexual behavior is influenced by psychosocial and cultural aspects and is not solely determined by biological factors. Literature on hypersexuality and problematic pornography consumption repeatedly found self-reported feelings of guilt, shame, or perceived wrongdoing in study participants. A phenomenon called “masturbatory guilt” has been described in individuals. Individuals who masturbate but on the other hand despise masturbation as morally reprehensible (Grubbs et al., 2019b) experience feelings of guilt. Superstition and ostracism have been banned from the general scientific discourse. However, it may persist in day-to-day behavior and thoughts of laypersons. Although societal attitudes have fundamentally changed, masturbation still remains a supplanted and tabooed topic for many. Negative effects of masturbation are caused by feelings of guilt, moral attitudes, and religious beliefs and not by the behavior itself, for which no ill effects have been found (Coleman, 2003). Pivotal for negative health effects of masturbation is a subjective evaluation of the behavior and its accompanying physical reactions. Massive guilt is experienced by some individuals, which then, in turn, influences psychological and relational well-being. One study (Castellini et al., 2016) reported that this so-called ego-dystonic masturbation was significantly related to higher scores of anxiety and depression scales, sexual dysfunctions, and relational as well as intrapsychic problems in a sample of over 4,000 human male outpatients of an andrology and sexual medicine clinic in Italy. The results indicate that masturbation seems to be a common behavior even in individuals with negative attitudes toward it. In their study, intra- and interindividual psychological strain was caused by the moral attitudes that were in conflict with the individual behavior. One study (Chakrabarti et al., 2002) portrays a man in a single-case study, who developed a depression of clinical extent on the ground of masturbatory guilt. He was successfully treated by providing education and information about human male sexuality and masturbation. Although this case study can only function for illustrative purposes, it underlines to which extent beliefs might mediate the relation between masturbation and health.

A theoretical framework that incorporates moral beliefs, norms, and personal attitudes has been proposed (Grubbs et al., 2019a, b). It provides a to-be-tested idea of how masturbatory behavior generally impacts mental health. The authors (Grubbs et al., 2019b) promote the idea that not the behavior itself or its frequency is the driving force in the reported psychological strain but its moral evaluation. Hence, self-reported psychological difficulties might be understood as an expression of moral incongruence: a discrepancy between beliefs and behavior. The theoretical framework was tested in exploratory studies in the area of pornography consumption, reporting that the strongest predictors of self-reported pornography addiction are religiousness and moral incongruence (Grubbs et al., 2019a). We believe that the framework could be suitably applied to masturbation accompanied by negative feelings.

Another study (Zimmer and Imhoff, 2020) examined the motivation for abstinence in a large, online-based survey. The authors found that the motivation to abstain from masturbation most strongly correlated with attitudes toward masturbation. Expected negative impact of masturbation, religiosity, conservatism, and lower trust in science were related to the motivation to abstain. This study validates the idea that the relation between masturbation and negative effects could be mediated by personal attitudes and beliefs. Self-reported excessive masturbation or even addictive behavior have been documented as cause for distress. Studies also found penile insensitivity, sometimes less satisfying partnered sex, and often an association with pornography consumption (Park et al., 2016; Dwulit and Rzymski, 2019). Studies addressing compulsive sexual behavior describe compulsive masturbation as one dimension of the self-reported behavior causing distress (Raymond et al., 2003; Kaplan and Krueger, 2010). Those studies show that masturbation contributes to stress in individuals reporting hypersexual behavior. Individuals that report compulsive masturbation often describe the frequency as problematic. Hence, the distress might truly be a dose-dependent effect.

That behavior patterns can cause distress when excessively practiced, in particular when a lack of control is perceived will be acknowledged in the ICD-11 in its category of compulsive sexual behavior disorder (Kraus et al., 2018; Fuss et al., 2019). Kafka (2010) reviewed the epidemiological literature on frequency of sexual behavior to define and propose diagnostic criteria for hypersexuality for the DSM-5, but the diagnosis was not included. Kafka (1997) proposed a total sexual outlet (TSO, orgasms per week, and independent of sexual activity) of 7 or more orgasms/week for a minimum duration of 6 months (Atwood and Gagnon, 1987; Långström and Hanson, 2006) as quantifiable criterium for hypersexuality. However, this criterium is still under debate (Moser, 2011; Wakefield, 2012). Besides the described difficulties, masturbation exhibits a plethora of positive effects on both mental and physical health (Mercer et al., 2005; Robbins et al., 2011; Mercer et al., 2013). It is commonly incorporated into sex therapy (LoPiccolo and Lobitz, 1972) and supports the development of comfort with one’s own body (Regnerus et al., 2017). Attempts to foster the development of a healthy sexuality explicitly incorporate masturbation as normal behavior. It is described as a powerful way to increase both immediate well-being and general sexual health and comfort. Masturbation is also promoted as a safe-sex behavior (Robinson et al., 2002; Coleman, 2003) to prevent the transmission of diseases. Some empirical studies found that masturbation reduces the risk of prostate cancer (Leitzmann et al., 2004; Aboul-Enein et al., 2016; Rider et al., 2016), although not completely conclusive [e.g., see Dimitropoulou et al. (2009)]. For the causal understanding of the potential relation between masturbation and prostate cancer risk, experimental, and longitudinal studies are needed.

In what follows, we now discuss the most important pending issues associated with masturbation and ejaculation frequency from the perspective of psychology and neuroscience, including accompanying methodological challenges. We emphasize that our ideas are broad topics of potential importance and not specific outlines of studies that are ready for implementation.

## Discussion

Pushing the idea that attitudes and beliefs mediate a potential relation between masturbation and negative feelings, creates a need for understanding the causal relation between both constructs. It is inherent to correlational studies that no causal relationship can be established. As masturbation has a long-standing history of superstition and ostracism up until today (Brody et al., 2012; Hoseini, 2017), causal studies are essential. Without the possibility for causal interpretation, results remain open to the respective theoretical background individuals hold. Although this argument is vital for any researcher in any field, it seems of special importance in an area where (a) not enough evidence-based knowledge exists at present and (b) social tabooing is still part of the phenomenon. Studies reporting a seemingly direct relation between masturbatory frequency and (mental) health wrongly derive causal conclusions from correlational data (Jiao et al., 2019), which is highly contestable. Case-control experiments and longitudinal data are needed to establish a substantial causal relation. As an area of research strongly influenced by cultural and social norms, the notion of studies representing WEIRD populations (Henrich et al., 2010) becomes an essential aspect.

Scientifically, masturbation is often addressed as a secondary aspect within areas of reproductive medicine, epidemiological studies on general sexual activity, or sexual disorders [see e.g., Wellings et al. (1990, 2006)]. However, up to date, research specifically addressing masturbation as the phenomenon of interest in itself remains an underrepresented aspect. Also, the concept of masturbation lacks a precise definition (Motofei and Rowland, 2005; Alwaal et al., 2015; Kirschbaum and Peterson, 2018). In an attempt to describe concepts held by the population about what behavior classifies as masturbation, Kirschbaum and Peterson (2018) it was found that participants held different notions of masturbation including a range of behaviors and situations (Atwood and Gagnon, 1987). Yet, “having an orgasm” and “being alone” were significant common denominators. The authors conclude that the vagueness of the definition, with only the aspects “orgasm” and “alone” as common denominators, mirrors a social discourse that is lacking an open discussion. Hence, behaviorally specific language is recommended for research on masturbation, as the term *per se* leaves room for interpretation. Explicitly defining, whether behavior solely in conjunction with orgasm or a broader scope of behaviors are addressed, should be a minimal requirement.

An interesting fundamental research direction would be the comprehensive examination of if and how masturbation affects quality of sperm parameters. Reproductive medicine has intensively examined aspects of sperm quality. While this is perfectly appropriate for understanding fertility difficulties, it does not fit for a derivation of knowledge concerning healthy populations. It is also not suited for the study of the effects on general or mental health. The selectivity of the sample makes it difficult if not impossible to isolate idiosyncrasies due to infertility from general aspects. Hence, results from reproductive medicine are revealing, however, impose a limitation on the transferability to the general population. It is difficult to design experimentally controlled studies in healthy individuals. Ethical restrictions make the intentional manipulation of sperm quality challenging or even unlawful. From our point of view, more well-designed studies examining masturbation as a vital and active part of healthy sexual activity and development across the lifespan are needed. Studies that systematically assess masturbatory behavior longitudinally and its potential changes or differential (age) impacts are lacking. No study has come to our attention that, for example, longitudinally addresses masturbatory behavior and potential effects in older age or in adults’ reproductive age along developmental trajectories. Some studies have addressed sexual activity within the context of successful aging and potential relations with other central variables such as cognitive functioning (Wright et al., 2017, 2019; Allen, 2018), but masturbation is treated as a secondary variable. Studies including masturbation as a potential predictor for health outcomes (comparable to studies including partnered sex), would be an interesting field of research across all ages. Research on both, ejaculation frequency as well as masturbation and its relation to brain structure and function are needed. Studying brain functional as well as structural mechanisms involved and affected by masturbation and ejaculation in a healthy, non-clinical sample of individuals could provide important insight into the underlying mechanisms. Results could exhibit potential effects of frequent ejaculation on brain structure. Also they would enable the localization of brain structures that could be predictive of frequent masturbation and ejaculation. Potential mediating constructs such as moral incongruence and its relation to brain structure and function could be studied in more detail.

Those studies must suitably address the technical difficulties in the assessment of ejaculation with imaging techniques (Georgiadis et al., 2007). Besides technical difficulties, sample size could impose a special challenge for studies in this field. Successful recruitment must overcome both the challenge of the delicate matter itself and the demands on individuals participating in an imaging study. Limitations in study designs are not unique to our area of interest, but a problem often seen in experimental research. However, it presumably is of special importance in a field that is still subject to impression management, social desirability, and shame at the same time.

We encourage future research to address the probably mediating effect of attitudes (personal as well as societal), religious beliefs, and cultural norms on the relation between masturbation and mental health. Coleman (2003) already made this assessment almost 20 years ago; however, it still applies today. While no direct negative effects of masturbation on health emerged up to date, the indirect impact on mental health has been discussed. The relation between masturbation and distress in individuals experiencing a discrepancy between beliefs and attitudes and own behavior (i.e., moral incongruency, see Grubbs et al., 2019a, b), is in urgent need of a variety of well-designed and well-conducted studies. Existing studies reporting distress in combination with masturbatory behavior are either confounded by pornography consumption (Grubbs et al., 2019b) or examine individuals claiming to suffer from compulsive sexual behavior in general (Kaplan and Krueger, 2010). While this is an interesting and relevant aspect, we consider it equally important to understand the relation between frequency of masturbation, attitudes, and health in its own right. Understanding this relation could offer the opportunity to rebut stigmatization and prevailing against superstition.

Research addressing the question of mental health and masturbation is, indeed, still sometimes biased by a traditional and historical perspective on masturbation, making a scientific evaluation of results difficult (Brody and Costa, 2009; Brody et al., 2012; Hoseini, 2017; Jiao et al., 2019). While Zimmer and Imhoff (2020) critically discuss the fact that masturbatory guilt and distress is often particularly present in individuals not trusting scientific results, sometimes the scientific background itself is biased (Tashakori et al., 2017; Jiao et al., 2019). For the sake of providing a solid background against which teaching about masturbation and health effects is warranted, more unbiased, well-conducted studies are needed. Without evidence-based studies, the discussion remains opinion-based without a true right or wrong. Although this is true for any topic, we believe that it is especially important in an area ballasted with centuries of superstition and ostracism.

## Conclusion

The aim of the present paper was the attempt of reconciling existing literature on “effects of ejaculation on general and mental health.” To succeed at this ambitious task, we aimed at providing an overview and raising important questions that need to be answered. We also aimed at providing a background for which new projects could be designed, and, ultimately, proposing a direction for future research on this topic. We have learned from the literature that masturbation is a common behavior that falls within the normal range of healthy sexual activity. It is promoted as safe-sex behavior and applied in sex therapy (LoPiccolo and Lobitz, 1972; WHO Regional Office for Europe and BZgA, 2010). While this has been generally acknowledged in the scientific community, it is still subject to social tabooing and often marked with shame causing distress (Castellini et al., 2016). In behavioral and social sciences, research on implications of lifestyle variables for general and mental health is common. This is true for risk but also for everyday behavior such as physical activity, nutrition, and social interactions (e.g., Owen and Corfe, 2017; Warburton and Bredin, 2017; Alegría et al., 2018). It follows that systematically addressing the question of whether and how masturbation and ejaculation as a lifestyle variable are related to health and well-being represents a timely area of research.

## Author Contributions

AM, MR, and SK drafted the manuscript. AM, MR, SK, and JG conducted the research and contributed to and have approved the final manuscript. All authors contributed to the article and approved the submitted version.

## Conflict of Interest

The authors declare that the research was conducted in the absence of any commercial or financial relationships that could be construed as a potential conflict of interest.

## Publisher’s Note

All claims expressed in this article are solely those of the authors and do not necessarily represent those of their affiliated organizations, or those of the publisher, the editors and the reviewers. Any product that may be evaluated in this article, or claim that may be made by its manufacturer, is not guaranteed or endorsed by the publisher.

## Footnotes

1 An ambiguity of definitions exists for different terms used throughout the manuscript. Several related terms are used in the literature with each being used with a different emphasis while also often being applied interchangeably. Central terms are “ejaculation” (Moser, 2011) and “orgasm” (Wakefield, 2012), as well as total sexual outlet (TSO) which refers to orgasms per week, independent of sexual activity. “Masturbation” usually refers to sexual activities performed on oneself, typically focusing on stimulation of the genitals to orgasm (Meston and Frohlich, 2000), with different perceptions in lay people (Atwood and Gagnon, 1987). “Abstinence” describes the practice of refraining from some or all aspects of sexual activity. Spiritual texts often describe orgasm and ejaculation as two different entities and suggest men to learn to reach orgasms without ejaculation to not loose energy. The terms “sperm” and “semen” are also often used interchangeably although representing different biological entities. Semen describes the fluid of the ejaculate that carries the sperm out of the male body, sperm refers to the actual gamete. In the present paper we will not solve or further address the problem of definitions. We will mainly stick to “frequency of ejaculation,” however, will use different terms, if used in respective studies or contexts.
